# Incidence and Outcomes of Desmoplastic Small Round Cell Tumor: Results from the Surveillance, Epidemiology, and End Results Database

**DOI:** 10.1155/2014/680126

**Published:** 2014-11-05

**Authors:** Christina K. Lettieri, Pamela Garcia-Filion, Pooja Hingorani

**Affiliations:** ^1^Children's Hospital and Medical Center, Omaha, University of Nebraska Medical Center, 8200 Dodge Street, Omaha, NE 68114, USA; ^2^Phoenix Children's Hospital, 1919 E. Thomas Road, Phoenix, AZ 85006, USA

## Abstract

Desmoplastic small round cell tumor (DSRCT) is a rare but highly fatal malignancy. Due to the rarity of this neoplasm, no large population based studies exist. *Procedure*. This is a retrospective cohort analysis. Incidence rates were calculated based on sex and ethnicity and compared statistically. Gender-, ethnicity-, and treatment- based survival were calculated using the Kaplan-Meier method. *Results*. A total of 192 cases of DSRCT were identified. Peak incidence age was between 20 and 24 years. Age-adjusted incidence rate for blacks was 0.5 cases/million and for whites was 0.2 cases/million (*P* = 0.037). There was no statistically significant difference in survival based on gender or ethnicity. When adjusted for age, there was no statistically significant difference in survival amongst patients who received radiation therapy compared to those who did not (HRadj = 0.73; 95% CI 0.49, 1.11). There was a statistically significant survival advantage for patients who received radiation after surgery compared to those who did not (HR 0.49; 95% CI 0.30, 0.79). *Conclusion*. DSRCT is more common in males and in people of African-American descent. Although overall survival remains poor, radiation therapy following surgery seems to improve outcome in these patients.

## 1. Introduction

Desmoplastic small round cell tumor (DSRCT) is a highly aggressive, rare tumor of mesenchymal origin whose oncogenic effect is presumed to originate from the unique chromosomal translocation t(11;22)(p13:q12) [1, 2], leading to the fusion of the N-terminal domain of Ewing's sarcoma gene* EWS*, to the C-terminal domain of Wilms' tumor suppressor gene,* WT1*, which is found in most but not all DSRCTs [3]. It tends to grow along a serosal lining, most commonly the peritoneal surface, but other primary sites have been described [4–7]. The typical histology includes variably sized clusters of small round or spindled cells lying within a desmoplastic, collagenous stroma [6]. No large population data exists regarding the epidemiology and best treatment of this tumor due to its rarity. Studies have shown that aggressive and complete surgical resection is a major determinant in patient survival [4, 8–12]; however, since DSRCT most commonly presents as a multicentric abdominal mass, complete upfront resection is not often possible. DSRCTs are chemosensitive, but often recur, necessitating multimodality therapy with radiotherapy, surgery, and/or high dose chemotherapy (HDCT) with stem cell rescue. Although many strategies have been attempted, survival in patients with DSRCT remains dismal.

## 2. Methods

SEER registry of the National Cancer Institute (NCI) is a well-reputed source of incidence, prevalence, and cancer survival statistics currently covering 28% of the United States population [13]. SEER database, SEER 17 Regs Research Data + Hurricane Katrina Impacted Louisiana Cases, was queried for all DSRCT cases from 1973 to 2007 using the ICD-O-3 = 8806/3 code. This database was used to obtain the largest possible cohort. Cases of DSRCT were selected based on the Hist/Behav (malignant) code 8806/3. The frequency session was used to ascertain the primary site and treatment by radiation and/or surgical treatment. Data on chemotherapy use were not available. Radiation (external beam or method NOS) was dichotomized as absent or present (at any time during therapy). There were no data available for radiation dosage. Surgery was recorded in SEER as “no cancer directed surgery or surgery in combination with radiation.” No data were available on outcome of patients with surgery alone and the extent of surgical resection.

Data were analyzed using the SEER∗Stat program 7.0.5., Stata SE 13.0 (College Station, TX), and GraphPad Prism Software version 5.04 (La Jolla, CA). The rate session of SEER∗Stat program 7.0.5 generated the overall gender- and ethnicity-based age-adjusted incidence rates standardized to the 2000 United States census population count. Age-adjusted incidence rates were calculated and compared between gender and ethnicity groups. Among cases with a known survival time, gender-, ethnicity-, and treatment-based survival curves were generated using the Kaplan-Meier method in GraphPad Prism Software. Cox regression models were constructed to estimate the hazard ratio for the association of race and radiation therapy with survival at 5 years. For radiation therapy, two models were constructed and adjusted for age at diagnosis: radiation at any time and radiation following surgical resection. Hazard ratios (HR) are presented with a 95% confidence interval (CI). Statistical significance was defined as alpha less than 0.5 with two-sided alternative hypotheses.

## 3. Results

From the SEER database, 192 cases (age 0–60 years) of DSRCT were identified. The common primary site(s) of disease were the peritoneum or soft tissue of abdomen and pelvis. Less common primary sites included the ovary/fallopian tube (6 cases), orbit (1 case), cerebellum (1 case), and cerebral ventricle (1 case) (Table 1).

**Figure 1:**
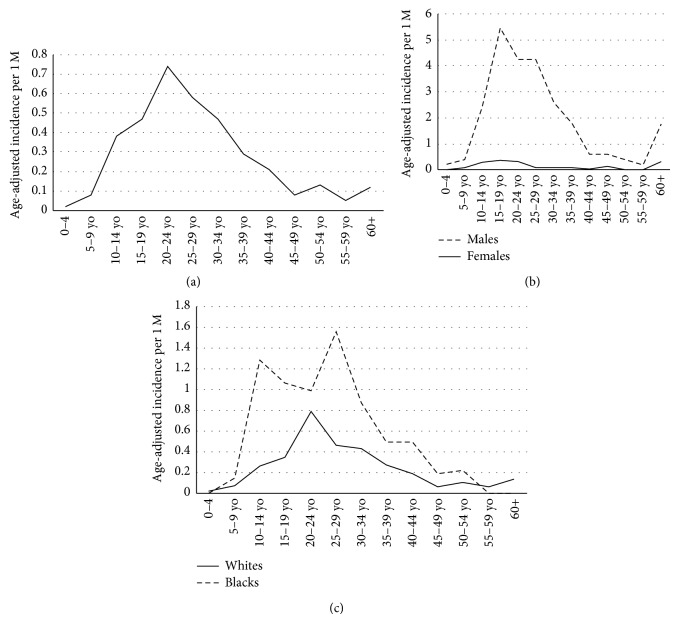
(a) Overall age-adjusted incidence of DSRCT. (b) Sex-based age-adjusted incidence of DSRCT. Males are more likely than females to get DSRCT (*P* < 0.001). (c) Race-based, age-adjusted incidence of DSRCT. Blacks are more likely than whites to get DSRCT (*P* = 0.037).

The age-adjusted incidence rate of DSRCT was 0.3 cases/million, with a peak incidence of 0.74 cases/million in persons 20–24 years of age (Figure 1(a)). In blacks, the peak incidence was seen in the age range of 25–29 years (Figure 1(c)). Age-adjusted incidence rates for males and females were 0.4 and 0.1 cases/million, respectively (*P* < 0.001) (Figure 1(b)). Age-adjusted incidence rates for Caucasians, African-Americans, American Indians, and Asian Pacific Islanders were 0.2, 0.5, 0.3, and 0.1 cases/million, respectively (data not shown), with a statistically significant difference between Caucasians and African-Americans (*P* = 0.037) (Figure 1(c)).

**Figure 2:**
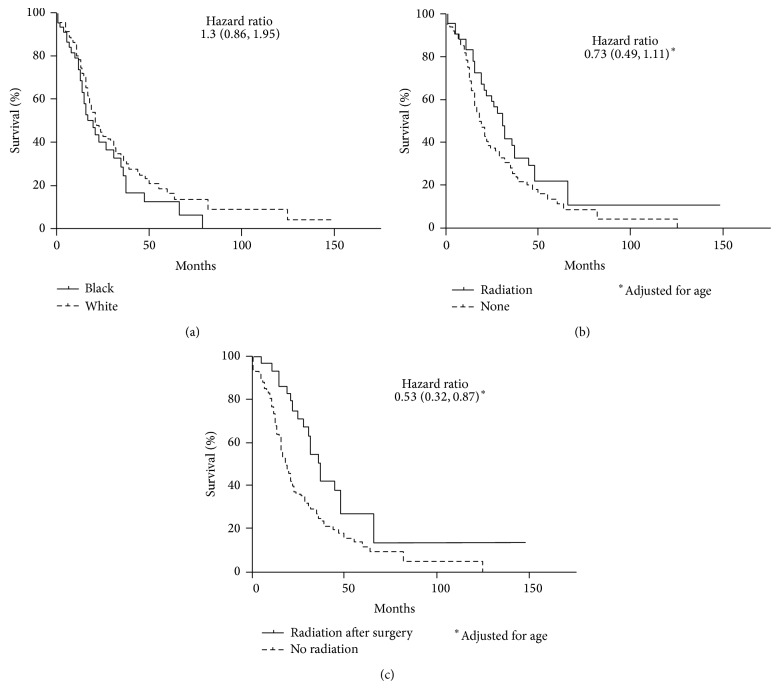
(a) Race-based survival of DSRCT. There may be a survival disadvantage for blacks compared to whites. Although it did not reach statistical significance, this analysis suggests that blacks are 33% more likely to succumb to DSRCT than are whites (*P* = 0.2). (b) Treatment-based survival of DSRCT, radiation versus no radiation. There was no statistically significant difference in survival amongst patients who received radiation therapy compared to those who did not. (c) Treatment-based survival of DSRCT, radiation after surgery versus no radiation. Patients who received radiation following surgery fared better than those patients who did not (*P* < 0.05).

The overall 5-year survival was 33.3%. There was no difference in 5-year survival between patients of 18 years and younger (30.9%) and over 18 years (34%). There was also no difference in survival between males and females. Compared to Caucasian cases, death was 30% more likely among African-American cases (HR: 1.30; 95% CI: 0.86, 1.95) (Figure 2(a)). After adjusting age at diagnosis, radiation therapy at any time was not associated with improved survival (HR_adj_ = 0.73; 95% CI 0.49, 1.11) (Figure 2(b)). Radiation administered following surgical resection improved survival at 5 years (HR_unadj_: 0.49; 95% CI 0.30, 0.79) (Figure 2(c)). The statistically significant association remained after adjusting for age at diagnosis (HR_adj_ 0.53; 95% CI 0.32, 0.87).

## 4. Discussion

This is the first SEER database analysis evaluating both incidence and survival data among patients with DSRCT. The majority of published data on DSRCT are single institution studies analyzing small numbers of cases given heterogeneous treatment. SEER data is inherently limited due to the voluntary nature of data collection. The diagnostic process is done locally and information including immunohistochemistry or molecular confirmation of the diagnosis is not available. The analysis of a rare event such as DSCRT must be analyzed from data that is gathered over a long time period and is subject to variability from different suppliers of data. One such limitation is the lack of information on dose or format of radiation therapy given. Also data on specific chemotherapy agents, doses, and schedules are not available in this data set. In addition, the SEER database does not provide data on recurrences. Summarization of end results is imperfect given these limitations; however, the data collected can be useful to provide insight and direct future investigations on patient outcomes.

This study is consistent with previous reports in that DRSCT was found to be more prevalent in males [4, 6, 10–12] and African-Americans [14]. Male to female ratio is 4 : 1 and similar to previously reported [3, 15]. Although the survival differences between blacks and whites suggest that blacks are 33% more likely to die of disease progression at 5 years than whites, this trend did not reach statistical significance likely secondary to small sample size. Disparities in socioeconomic status and availability of medical care are risk factors in the ethnic minority populations which are known to result in worse outcomes in several disease types including pain management [16], depression [17], and cancer [18]. Whether such differences influence outcome in DSRCT or whether there are differences in biology of this tumor between various ethnic groups remains unknown. Without large prospective clinical trial data, standard of care for treatment of DSRCT has not been established. As previously described [9], patients who received radiation therapy did not show a statistically significant survival advantage compared to patients who had not received radiation therapy. There was, however, a statistically significant difference in survival for patients who received radiation after surgery compared to patients who received no radiation. This is consistent with other studies [12, 19]. The patients described in the literature with the longest survival tend to have received multimodality therapy including aggressive surgery, multiagent chemotherapy including hyperthermic peritoneal perfusion, and radiation therapy [20]. This review of SEER data supports inclusion of multimodality therapy in treatment for DSRCT, with data revealing improvement in survival by 32% compared to those without radiation treatment. Overall survival however remains poor and the need for additional novel targeted therapies for this rare tumor is paramount.

## Figures and Tables

**Table 1:** Demographics of DSCRT.

Demographic	Number	% total
% male	150	78.13
% female	42	21.88
Age		
0–9 yrs	6	3.13
10–19 yrs	43	22.40
20–29 yrs	67	34.90
30–39 yrs	39	20.31
40–49 yrs	16	8.33
50–59 yrs	8	4.17
60+ yrs	13	6.77
≤18 yrs	42	21.88
>18 yrs	150	78.13
Ethnicity		
White	134	69.79
Black	46	23.96
Other	12	6.25
Tumor location		
CNS/orbit	4	2.08
Facial (includes sinuses, nasal cavity, and conn tissue)	4	2.08
GI tract-stomach	3	1.56
GI tract-jejunum through rectum	7	3.65
Lung (bronchi or lobe)	3	1.56
Lower limb	2	1.04
Upper limb	1	0.52
Kidney or retroperitoneum	11	5.73
Abdomen: connective or nervous tissue	90	46.88
Pelvis: connective or nervous tissue	26	13.54
Ovary/fallopian tube	6	3.13
Prostate	1	0.52
Connective tissue, NOS or trunk	17	8.85
Overlap peripheral nerves, autonomic nervous system, NOS	1	0.52
Unknown	16	8.33
